# Serologic Evidence of Exposure to *Burkholderia pseudomallei*, Nigeria

**DOI:** 10.3201/eid3201.251113

**Published:** 2026-01

**Authors:** Jelmer Savelkoel, Gabriel E. Wagner, Chiedozie K. Ojide, Katrin Frankenfeld, Anne Rudloff, Susanna J. Dunachie, Michaela Lipp, W. Joost Wiersinga, Ivo Steinmetz, Emma Birnie, Rita O. Oladele

**Affiliations:** Amsterdam UMC location University of Amsterdam, Amsterdam, the Netherlands (J. Savelkoel, W.J. Wiersinga, E. Birnie); Medical University of Graz, Graz, Austria (G.E. Wagner, M. Lipp, I. Steinmetz); Ebonyi State University, Abakaliki, Nigeria (C.K. Ojide); Alex Ekwueme Federal University Teaching Hospital, Abakaliki (C.K. Ojide); Research Center for Medical Technology and Biotechnology, fzmb GmbH, Bad Langensalza, Germany (K. Frankenfeld, A. Rudloff); Mahidol-Oxford Tropical Medicine Research Unit, Mahidol University, Bangkok, Thailand (S.J. Dunachie); University of Oxford, Oxford, UK (S.J. Dunachie); College of Medicine University of Lagos, Lagos, Nigeria (R.O. Oladele); Lagos University Teaching Hospital, Lagos (R.O. Oladele)

**Keywords:** *Burkholderia pseudomallei*, bacteria, melioidosis, Nigeria, serology, epidemiology

## Abstract

Melioidosis is an underreported cause of community-acquired pneumonia and sepsis in Nigeria. We conducted a cross-sectional study using a *Burkholderia pseudomallei* protein microarray in 500 healthy participants from Nigeria. We observed a serologic response supportive of past exposure to the causative agent of melioidosis in 30% of study participants.

Melioidosis is a severely neglected tropical disease that is prevalent yet highly underreported in Africa (*1*,*2*). *Burkholderia pseudomallei*, as its causative agent, is a gram-negative environmental bacterium, and exposure can lead to pneumonia, sepsis, and abscess formation; mortality rates sometimes exceed 40% (*1*). Nigeria is a high-priority country for melioidosis surveillance efforts in Africa because the disease burden is thought to the highest on the continent (*2*–*4*). However, current epidemiologic estimates in Nigeria are derived solely from modeling (*3*,*4*). Environmental distribution of *B. pseudomallei* has been established in Nigeria; the highest soil positivity rate has been demonstrated in the southeastern state Ebonyi (*5*). We hypothesized that environmental exposure to *B. pseudomallei* would result in a serologic response among inhabitants of Ebonyi state. Thus, we performed a cross-sectional study to assess the extent of a serologic response to *B. pseudomallei* in this state. 

We recruited healthy participants using a convenience sampling approach in communities and the blood bank of the Alex Ekwueme Federal University Teaching Hospital Abakaliki. Adult participants were only able to take part if they could provide consent and were without suspicion of acute disease or febrile illness. Every consecutive participant meeting those criteria was recruited using a 1:1 ratio between communities and the blood bank. We obtained serum samples, and participants completed a short survey with questions on demographics and risk factors related to *B. pseudomallei* exposure. 

We tested serum at a 1:2,000 dilution for the presence of IgG against a panel of 4 *B. pseudomallei* antigens (BPSL2096, BPSL2697, BPSS0477, and BPSS1498) spotted at a concentration of 1 mg/mL on the INTER-ARRAY platform (fzmb GmbH, https://www.inter-array.com), and we applied a signal intensity threshold of 0.3 on the basis of previous studies (*6*,*7*) (Appendix). We verified array performance with buffer control and nonpooled positive (melioidosis, culture-confirmed) and negative (healthy, nonendemic) control samples in every run. We screened serum of healthy participants from the hyperendemic region of Ubon Ratchathani, Thailand, collected as part of a previous study, in parallel with the same assay and used it as a comparator (n = 50) (*6*,*8*).

Ethical approval for use of the Nigeria samples was obtained from the Research and Ethics Committee of Alex Ekwueme Federal University Teaching Hospital Abakaliki (ref. no. 10/06-2022-02/08/2022). Ethical approval for the use of the Thailand samples was obtained from the Faculty of Tropical Medicine, Mahidol University (ref. no. TMEC 12-014), Sappasitthiprasong Hospital, Ubon Ratchathani (ref. no. 018/2555), and the Oxford Tropical Research Ethics Committee (ref. no. 64-11); study details can be found elsewhere (*6*,*8*).

We recruited 500 participants in Nigeria, 60.6% men and 39.4% women, with a median age of 30 (interquartile range 23–38) years; most reported farming activities and soil exposure as risk factors for exposure to *B. pseudomallei* (Table). We observed 150 (30%) participants with a positive serologic response to *>*1 antigen of our panel, compared to 23 (46%) participants for the Thailand cohort (Figure). We observed positivity to BPSS1498, also known as hemolysin coregulated protein and considered to be the prime serodiagnostic target for *B. pseudomallei* and *B. mallei* (*7*), in 82 (16.4%) participants from Nigeria (Table; Figure, panel A). In Thailand, 20 (40%) participants had a positive response to BPSS1498 (Figure, panel B). No participants from Nigeria had a positive response to >3 antigens. We included the variables age, sex, smoking and alcohol use, soil exposure and farming activity, and sampling location (community vs. blood bank) in a logistic regression model. However, we excluded diabetes from this model because of the limited number of events, despite it being a known risk factor for melioidosis (*1*). Using the previously mentioned model, we found no significant predictors of a positive serologic response in participants from Nigeria when taking into account all pooled antigens.

**Table:** Demographic characteristics and serologic responses of 500 healthy participants in Ebonyi state, Nigeria, in study of serologic evidence of exposure to Burkholderia pseudomallei*

Variable	Participants, n = 500
Sex	
M	303 (60.6)
F	197 (39.4)
Median age, y (IQR)	30.00 (23.00–38.00)
Farmer	370 (74.0)
Barefoot soil exposure	306 (61.2)
Smoking, current	32 (6.4)
Alcohol use, current	243 (48.6)
Diabetes	4 (0.8)
Serologic response	
BPSL2096	110 (22.0)
BPSL2697	2 (0.4)
BPSS0477	0
BPSS1498	82 (16.4)

*Values are no. (%) except as indicated. IQR, interquartile range.

**Figure:**
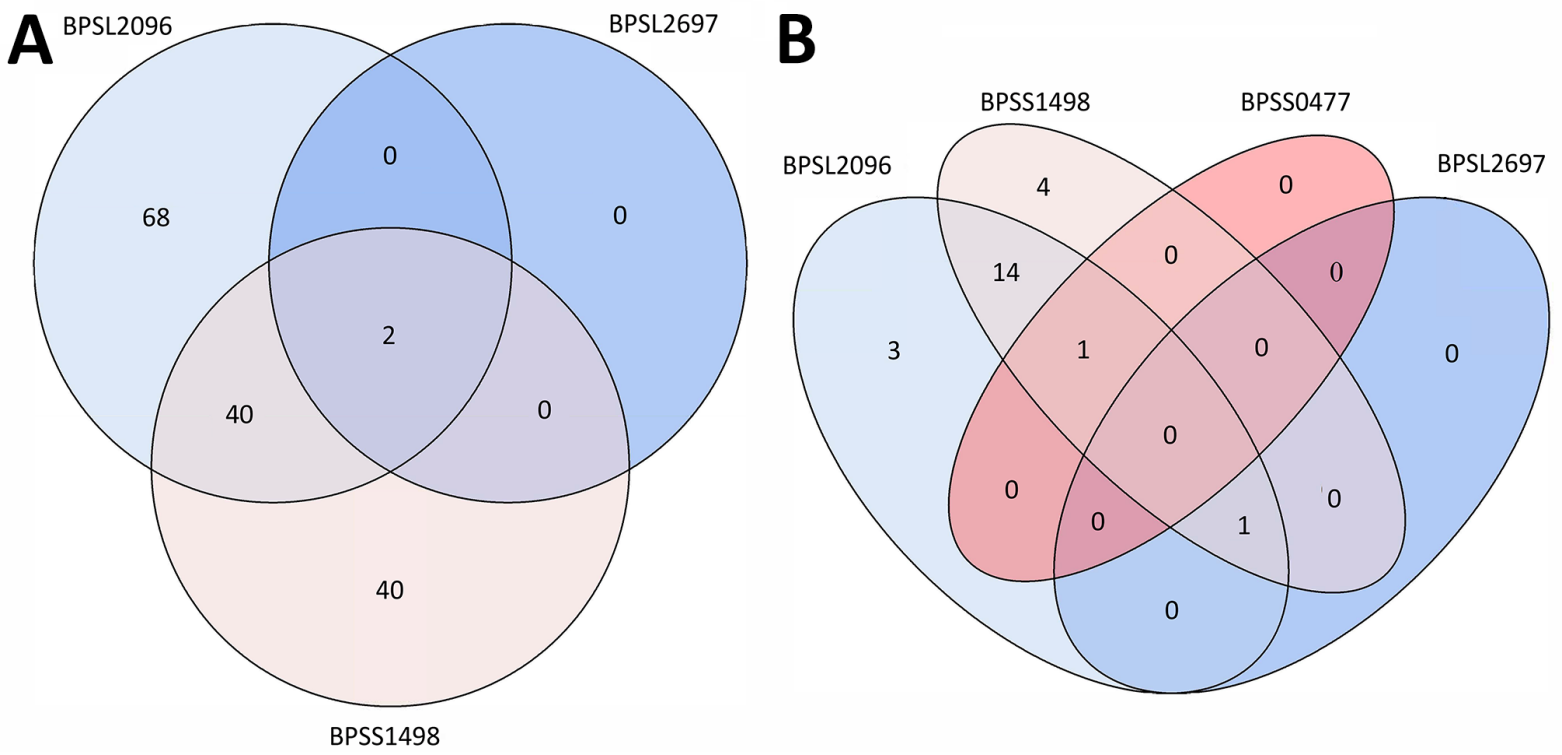
Venn diagrams displaying the serologic responses of participants from Nigeria (A) and Thailand (B) from study of serologic evidence of exposure to *Burkholderia pseudomallei*, Nigeria. The Venn diagram illustrates participants with a serologic response to >1 antigens (BPSL2096, BPSL2697, BPSS0477, and BPSS1498). Antigen BPSS0477 was excluded from the Nigeria Venn diagram because no participants displayed a positive response.

Strengths of our study include the use of a multiplex assay that includes BPSS1498, which alone proves more sensitive as a serodiagnostic target than the indirect hemagglutination assay, the standard for melioidosis serodiagnosis (*9*,*10*). Of note, BPSL2096 showed a higher positivity in participants from Nigeria than BPSS1498 did, which might reflect differences in bacterial strains, host factors, or both. However, further studies are needed to elucidate this observation. Our positive results are strengthened by the fact that we tested serum from participants from regions in Nigeria in which the pathogen has been detected in the environment. Also, we compared our results to a selection of healthy serum samples from an endemic area in northeastern Thailand to contextualize our results. Limitations include possible underestimation of the seropositivity rate because of the use of a serodiagnostic cutoff value for signal intensity established in culture-confirmed melioidosis patients (*6*) or overestimation because of possible but considered limited cross-reactivity after exposure to other species (*9*,*10*). 

Our seroepidemiologic panel should be further refined relating to cutoffs in ongoing surveillance efforts in Nigeria and other sub-Saharan countries to map the epidemiology of melioidosis. Our seropositivity estimates of exposure to *B. pseudomallei* among persons in Nigeria can be used to inform future serosurveillance and validation work.

Appendix Additional information about serologic evidence of exposure to *Burkholderia pseudomallei*, Nigeria.
