# Supplementary material for: Serologic Evidence of Exposure to Burkholderia pseudomallei, Nigeria
Source: Emerg Infect Dis. 2026 Jan;32(1):156–8. doi: 10.3201/eid3201.251113 (PMC12870078; doi:10.3201/eid3201.251113)
Supplement: Appendix — Additional information about serologic evidence of exposure to Burkholderia pseudomallei, Nigeria. [file 25-1113-Techapp-s1.pdf]

Article DOI: https://doi.org/10.3201/eid3201.251113

EID cannot ensure accessibility for supplementary materials supplied by authors. Readers who have difficulty accessing supplementary content should contact the authors for assistance.

Serologic Evidence of Exposure to *Burkholderia pseudomallei*, Nigeria

Appendix

Serologic *B. pseudomallei* protein microarray protocol

Testing of the sera with the *B. pseudomallei* protein microarray (fzmb GmbH, https://www.inter-array.com/) was carried out according to the manufacturer's instructions using a thermoshaker and the buffers mentioned below. In short, arrays were washed twice with array buffer (150 µl, 5 min, 400 rpm, 37°C), blocked with blocking buffer (100 µl, 5 min, 300 rpm, 37°C) and incubated with serum diluted 1:2,000 in array buffer (100 µl, 30 min, 300 rpm, 37°C). Subsequently, microarrays were washed once with array buffer (150 µl, 5 min, 400 rpm, 37°C), incubated with horseradish peroxidase-conjugated anti-human IgG antibody (Sigma-Aldrich, https://www.sigmaaldrich.com) diluted 1:667 in array buffer (100 µl, 30 min, 300 rpm, 37°C), washed twice in array buffer (150 µl, 4 min, 400 rpm, 37°C) and incubated with substrate (100 µl, 10 min, no shaking, room temperature) (Seramun Diagnostica GmbH, https://www.seramun.com). The array buffer consists of 1× phosphate-buffered saline, 0.05% Tween 20 and 0.25% Triton. Blocking buffer consists of 2% milk powder (Carl Roth, https://www.carlroth.com) dissolved in array buffer.
